# Evaluating sensitivity to classification uncertainty in latent subgroup effect analyses

**DOI:** 10.1186/s12874-022-01720-8

**Published:** 2022-09-24

**Authors:** Wen Wei Loh, Jee-Seon Kim

**Affiliations:** 1 Department of Data Analysis, Ghent University, Gent, Belgium; 2 Department of Quantitative Theory and Methods, Emory University, Atlanta, GA USA; 3 Department of Educational Psychology, University of Wisconsin–Madison, Madison, Wisconsin USA

**Keywords:** Causal inference, Finite mixture models, Latent class analysis, Parametric bootstrap, Perturbed confidence interval, Sensitivity analysis, Subgroup average treatment effect (ATE), 62D20, 62H30, 62P25

## Abstract

**Background:**

Increasing attention is being given to assessing treatment effect heterogeneity among individuals belonging to qualitatively different latent subgroups. Inference routinely proceeds by first partitioning the individuals into subgroups, then estimating the subgroup-specific average treatment effects. However, because the subgroups are only latently associated with the observed variables, the actual individual subgroup memberships are rarely known with certainty in practice and thus have to be imputed. Ignoring the uncertainty in the imputed memberships precludes misclassification errors, potentially leading to biased results and incorrect conclusions.

**Methods:**

We propose a strategy for assessing the sensitivity of inference to classification uncertainty when using such classify-analyze approaches for subgroup effect analyses. We exploit each individual’s typically nonzero predictive or posterior subgroup membership probabilities to gauge the stability of the resultant subgroup-specific average causal effects estimates over different, carefully selected subsets of the individuals. Because the membership probabilities are subject to sampling variability, we propose Monte Carlo confidence intervals that explicitly acknowledge the imprecision in the estimated subgroup memberships via perturbations using a parametric bootstrap. The proposal is widely applicable and avoids stringent causal or structural assumptions that existing bias-adjustment or bias-correction methods rely on.

**Results:**

Using two different publicly available real-world datasets, we illustrate how the proposed strategy supplements existing latent subgroup effect analyses to shed light on the potential impact of classification uncertainty on inference. First, individuals are partitioned into latent subgroups based on their medical and health history. Then within each fixed latent subgroup, the average treatment effect is assessed using an augmented inverse propensity score weighted estimator. Finally, utilizing the proposed sensitivity analysis reveals different subgroup-specific effects that are mostly insensitive to potential misclassification.

**Conclusions:**

Our proposed sensitivity analysis is straightforward to implement, provides both graphical and numerical summaries, and readily permits assessing the sensitivity of any machine learning-based causal effect estimator to classification uncertainty. We recommend making such sensitivity analyses more routine in latent subgroup effect analyses.

**Supplementary Information:**

The online version contains supplementary material available at 10.1186/s12874-022-01720-8.

## Introduction

Researchers in the behavioral, health and social sciences are increasingly interested in investigating how the causal effect of a treatment on an outcome differs among individuals in qualitatively different latent subgroups; see e.g., [1, 2, 3, 4, 5, 6, 7, 8, 9, 10]. For example, the effect of a medical intervention, such as right heart catheterization, on the six-month mortality of critically ill patients [11] may differ across patients with different latent risk profiles that depend on their medical and health status. But it is often impractical or impossible to measure all possible (baseline or pretreatment) covariates that are jointly associated with the latent subgrouping that characterizes treatment effect heterogeneity. Moreover, estimating fine-grained treatment effects moderated by all possible combinations of the observed covariates may be practically impossible due to the curse of dimensionality; and, even if possible, will likely lack adequate statistical power to detect distinct subgroup-specific effects.

An alternative approach is to first partition the individuals by exploiting the observed covariates’ associations with the latent subgrouping, then estimate the average treatment effect (ATE) within each imputed subgroup. For example, latent subgroups have been defined based on classes or components derived from observed covariates (and treatment) using either latent class models [12, 13, 14, 15, 16, 17, 18, 19], or Gaussian mixture models [20, 21], or mixture (zero-inflated) negative binomial regression models for (zero-inflated) count data [22], or mixture logistic regression models for treatment given covariates [23, 24, 25], or longitudinal growth patterns for classifying patients [26, 27, 28, 29]. Hence each individual’s (predictive or posterior) probabilities of belonging to each distinct latent subgroup are typically estimated using finite mixture models, such as (classical) latent class models [30, 31, 32, 33, 34, 35], model-based clustering [36], or finite mixture regressions [37]; see [38] for applications of such models in medical research.

### Limitations of existing methods

Regardless of how the latent subgroups are defined, under a “classify-analyze” approach (also known as “modal” or “hard” assignment), each individual’s imputed subgroup membership is determined simply as the subgroup for which their probability of membership is greatest. The resulting partitions are subsequently fixed when analyzing the subgroup-specific average causal effects. Therefore, each imputed subgroup can potentially be contaminated with individuals from a different subgroup, resulting in biased estimates and misleading substantive conclusions. Such classification errors arise even under an “expected-value” approach (also known as “proportional” or “soft” assignment) where each individual is assigned to every possible latent subgroup using fractional weights proportional to their membership probabilities [39, 40]. Because misclassification results from prediction and not sampling errors, the biases persist even in large samples or at the population level.^1^ Nonetheless, misclassification biases can be corrected under specific modeling and structural assumptions. For example, when using a latent class model, [13], following [41] and [42], derives unbiased estimators of the class-specific average treatment effect.

**Fig. 1:**
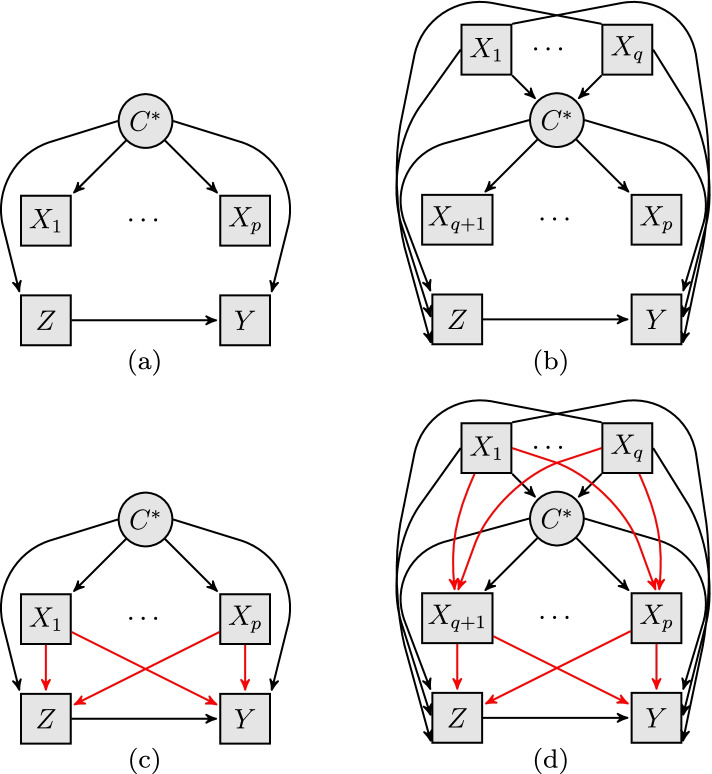
Causal diagrams depicting different causal (or structural) relationships between the indicators of the latent class $C^*$, and the “external” observed variables such as treatment (*Z*) and outcome (*Y*). In the left column (subfigures **a** and **c**), no covariates are predictors of the latent class, so $(X_1, \ldots , X_p)$ are indicators. In the right column (subfigures **b** and **d**), a subset of the covariates $(X_1, \ldots , X_q)$ are (explanatory) predictors of the latent class and its indicators $(X_{q+1}, \ldots , X_p)$. In the top row (subfigures **a** and **b**), the indicators are conditionally independent of all external variables given the latent class, as represented by the absence of arrows linking the indicators with any other observed variables. In the bottom row (subfigures **c** and **d**), the indicators are permitted to affect, or be affected by, external observed variables, as represented by the red arrows. Rectangular nodes denote observed variables, while round nodes denote latent variables

But such methods rule out any covariates associated with the (potential) outcomes from being indicators of the latent classes; see note 16 in Assumption 2 of [13]. One must therefore impose a structural assumption that latent class indicators be conditionally independent of all other observed variables given the latent class, such as in the causal diagrams of Fig. 1a and b. Such conditional independence between latent class indicators and “external” observed variables, that are not indicators of the latent class, is a standard assumption of basic latent class analysis [43] but is often violated in practice [44]. In practice, any of the indicators may simultaneously be causes of treatment or outcome, as represented by the red arrows in Fig. 1c and d, thus invalidating the bias-adjusted methods that are predicated on the absence of such effects. Recent modifications to accommodate effects between latent class indicators and external variables are limited to “one or two” ([45], p.361) external variables, which can be unrealistic when there are several covariates that directly affect the indicators, which in turn directly affect both treatment and outcome, such as in Fig. 1d. Under such complex settings, “one-step” estimation of the (joint) likelihood assuming a parametric model for all the variables is recommended [45]. But such parametric approaches rule out utilizing machine learning-based treatment effect estimators that are increasingly prevalent for causal inference [46, 47, 48]. Furthermore, existing bias-correction methods offer no insight into how the effect estimator’s sensitivity to classification uncertainty can systematically affect the subgroup effects.

### Proposed sensitivity analysis to classification uncertainty

Given the above shortcomings, in this paper, we propose a novel sensitivity analysis strategy that proceeds along different lines than existing methods that merely seek to correct for misclassification biases. In the first part, we exploit the (predictive or posterior) subgroup membership probabilities that reflect the quality and strength of evidence of the individual memberships to construct different (nested) subsets for each subgroup carefully. In the second part, the subgroup-specific average treatment effect estimates are then calculated for each different subset. The impact of classification uncertainty on the (in)stability of the effect estimates can then be inspected visually using graphical displays and assessed empirically using numerical summaries. Moreover, to acknowledge the inherent sampling uncertainty in the membership probabilities, we adopt a *parametric bootstrap* following [49] for inference about the subgroup effects.

Therefore, the proposal offers several attractive features by directly assessing and quantifying the impact of classification uncertainty on subgroup effect analyses. First, no restrictions on the causal or structural model, such as the absence of indicator-treatment and indicator-outcome relations, are imposed when defining the latent subgroups. Hence, all observed covariates may be used simultaneously as indicators of the latent subgroups and as conditioning variables for confounding adjustment. Second, any treatment effect estimator can be utilized without modeling assumptions, such as being compelled to include a statistical interaction term between the treatment and the latent subgroup under parametric outcome regression models. Hence, the treatment and outcome can be continuous or non-continuous, with non-continuous variables accommodated using non-linear models. We will demonstrate the procedure using a *doubly-robust augmented inverse propensity weighted (DR-AIPW)* estimator [50, 51, 52] that is endowed with attractive statistical properties and combines (parametric regression) models for the outcome given treatment and covariates and for the treatment given covariates. Third, any finite mixture model - including but not limited to latent class analysis - can be utilized to define (and measure) the latent subgroups that characterize treatment effect heterogeneity. Hence, indicators of the latent classes may be continuous, categorical, or a combination of both. Finally, existing bias correction methods can be readily incorporated simply by using the bias-corrected estimate as a benchmark for comparing the relative (in)sensitivity of the trajectory of the effect estimator. Therefore, the proposed sensitivity analysis strategy can offer researchers a more rigorous assessment of the stability of substantive subgroup effect analyses in the presence of classification uncertainty.

The remainder of this article is as follows. In Section 2 notation is introduced, and the subgroup-specific causal effect of interest and its estimator is defined. Complications arising from misclassifying individuals to the latent subgroups are described. In Section 3, the proposed sensitivity analysis to classification uncertainty is presented. In Section 4 the proposed methods are illustrated using two different publicly available datasets. A discussion of existing methods, and future directions of research, is provided in Section 5. All methods are implemented using the open-source statistical computing environment R [53]. Steps to implement the parametric bootstrap under each of two common finite mixture modeling approaches are presented in the Online Supplemental Materials. Scripts replicating the illustrations and the simulation studies are freely available online^2^.

## Potential outcomes framework for subgroup-specific average treatment effects

We first define the causal effects of interest using the potential outcomes framework and describe the DR-AIPW effect estimator. Let $Y_{i}(z)$ denote the potential outcome for individual *i* had they, possibly counter to fact, received treatment $Z=z$. Let $C_{i}^{*}$ denote the actual subgroup membership for individual *i*, where asterisks denote true (latent or unknown) values in this article. Let $\mathcal {C}$ denote the set of possible values for $C_{i}^{*}$; e.g., $\mathcal {C} = \{1,2\}$ when there are only two subgroups. The individuals can therefore be partitioned into $|\mathcal {C}|$ subgroups based on their values of $C_{i}^{*}$. Denote the vector of true subgroup memberships for all individuals by $\varvec{C}^{*}=(C_{1}^{*},\ldots ,C_{n}^{*})$. As with common stepwise latent class methods, we will assume that the number of subgroups $|\mathcal {C}|$ is known. When there are no a priori assumptions about the number of latent classes, the general recommendation for common latent class methods is a stepwise approach [54, 55]. Multiple (measurement) models for the latent class and its indicators only, each with a different number of latent classes, are first fitted to the observed data. The number of classes is then selected using comparative model parsimony criteria, such as the Akaike information criterion (AIC) [56], Bayesian information criterion (BIC) [57] or model entropy; as well as other substantive criteria; see e.g., [58] for practical advice. We also apply selected criteria later in the illustrations. The selected number of classes is then fixed when introducing other (external) observed variables, such as covariates that may affect the latent class (i.e., “explanatory variables”), and treatment and outcome that may be affected by the latent class (i.e., “distal outcomes”). In this paper, we focus on only the uncertainty due to possible misclassification and not the uncertainty due to model selection and identification. We, therefore, adopt the same prevalent practice, including the assumption that the latent subgroups are causally antecedent to treatment and outcome, as is common when using established stepwise latent class methods [43].

Define the subgroup-specific (average causal) effect among individuals belonging to the same subgroup $c \in \mathcal {C}$ as $\tau _{c} = \mathrm {E}\{Y(1)-Y(0) | C^{*}=c\}$, where the expectation is over the subpopulation of individuals actually belonging to subgroup *c*. When treatment is randomly assigned, the subgroup-specific effects can be unbiasedly estimated by the difference between the average observed outcomes in the two treatment groups, among individuals in each subgroup. But when treatment is non-randomly assigned, baseline common causes of the treatment and the outcome, henceforth termed *confounders*, induce spurious correlations between treatment and outcome. Pre-treatment, or baseline, covariates that include any potential confounders must thus be adjusted for to eliminate biases due to observed confounding [59]. In this paper, we will assume that adjusting for the observed covariates is sufficient to eliminate all associations between treatment and outcome due to confounders. In other words, there is no unmeasured confounding between the observed treatment *Z* and potential outcomes $\{Y(1), Y(0)\}$ within strata defined by unique levels of the observed covariates. Such an assumption is routinely made to identify causal effects of interest, and we discuss in Section 5 complications from relaxing this assumption. Under the above assumption of no unmeasured confounding, an unbiased estimator of the subgroup-specific effect $\tau _{c}$ can be obtained by conditioning on the observed covariates within each true latent subgroup. We describe such an estimator in the next section.

### DR-AIPW estimator

In this section, we describe the doubly-robust augmented inverse propensity weighted (DR-AIPW) estimator of the subgroup-specific effect $\tau _{c}$. Let $Z_{i}$ and $\varvec{X}_{i} = (X_{1i}, \ldots , X_{pi})$ denote the observed treatment and vector of *p* baseline covariates, respectively, for individual *i*. For a binary treatment, the propensity score [60] for an individual *i* in subgroup *c* is defined as the conditional probability of receiving treatment given the observed covariates; i.e., $\Pr (Z_{i}=1|\varvec{X}_{i},C_{i}^{*}=c)$. For notational simplicity, we henceforth denote the individual (subgroup-specific) propensity score by $p(\varvec{X}_{i},C_{i}^{*}=c)=\Pr (Z_{i}=1|\varvec{X}_{i},C_{i}^{*}=c)$. Let $\mathrm {I}(A)$ denote the indicator function that takes value 1 when event *A* occurs, or 0 otherwise. Define the *inverse propensity (score) weight* [61, 62] for individual *i* (in subgroup *c*) by:

1 $$\begin{aligned} W_{i}^{c}(\varvec{C}^{*}) = \mathrm {I}(C_{i}^{*}=c) \left\{ \frac{Z_{i}}{p(\varvec{X}_{i},C_{i}^{*}=c)}+\frac{1-Z_{i}}{1-p(\varvec{X}_{i},C_{i}^{*}=c)}\right\} . \end{aligned}$$

Let $m(Z_{i}, \varvec{X}_{i}, C_{i}^{*}=c) = \mathrm {E}(Y_{i}|Z_{i},\varvec{X}_{i},C_{i}^{*}=c)$ denote the assumed outcome regression model given treatment and covariates within the (true) subgroup $C_{i}^{*}=c$. Let $m_{c}^{*}=\sum \limits _{i=1}^{n} \mathrm {I}(C_{i}^{*}=c)$ denote the number of individuals in the (true) subgroup $C_{i}^{*}=c$. Following [51], the DR-AIPW estimator of $\tau _{c}$ is:

2 $$\begin{aligned}&\widehat{\tau }_{c}(\varvec{C}^{*}) \nonumber \\&= \frac{1}{m_{c}^{*}} \sum \limits _{i=1}^{n} \mathrm {I}(C_{i}^{*}=c) \left[ (2Z_{i}-1)W_{i}^{c}(\varvec{C}^{*})Y_{i} - \{Z_{i} - p(\varvec{X}_{i},C_{i}^{*}=c)\} \right. \nonumber \\&\left. \quad \times \left\{ \frac{1}{p(\varvec{X}_{i},C_{i}^{*}=c)}m(Z_{i}=1, \varvec{X}_{i}, C_{i}^{*}=c) + \frac{1}{1-p(\varvec{X}_{i},C_{i}^{*}=c)}m(Z_{i}=0, \varvec{X}_{i}, C_{i}^{*}=c) \right\} \right] . \end{aligned}$$

When both propensity score (PS) and outcome models are correctly specified, the variance of the estimator is consistently estimated by:

3 $$\begin{aligned} \widehat{\mathrm {V}}_{c}(\varvec{C}^{*})&= \frac{1}{m_{c}^{*}(m_{c}^{*}-1)} \sum \limits _{i=1}^{n} \mathrm {I}(C_{i}^{*}=c) IF_{i}(C_{i}^{*}=c)^{2};\end{aligned}$$

4 $$\begin{aligned} IF_{i}(C_{i}^{*}=c)&= \mathrm {I}(C_{i}^{*}=c) \left[ (2Z_{i}-1)W_{i}^{c}(\varvec{C}^{*})Y_{i} - \{Z_{i} - p(\varvec{X}_{i},C_{i}^{*}=c)\}\right. \nonumber \\&\quad \times \left\{ \frac{1}{p(\varvec{X}_{i},C_{i}^{*}=c)}m(Z_{i}=1, \varvec{X}_{i}, C_{i}^{*}=c) \right. \end{aligned}$$

5 $$\begin{aligned}&\quad \quad \left. + \left. \frac{1}{1-p(\varvec{X}_{i},C_{i}^{*}=c)}m(Z_{i}=0, \varvec{X}_{i}, C_{i}^{*}=c) \right\} \right] - \widehat{\tau }_{c}(\varvec{C}^{*}). \end{aligned}$$

A Wald $100(1-\alpha )\%$ confidence interval (CI) can be constructed by adding and subtracting the point estimate $\widehat{\tau }_{c}(\varvec{C}^{*})$ by the product of the $\alpha /2$ quantile of a standard normal distribution and $\sqrt{\widehat{\mathrm {V}}_{c}(\varvec{C}^{*})}$.

The DR-AIPW estimator is attractive because it is asymptotically unbiased when both the propensity score model and outcome model are correctly specified and consistent if only one model is correctly specified [50, 63]. Moreover, it permits reducing the reliance on routine parametric regression models that demand (correctly) specifying the exact relationships between the covariates and treatment, or outcome, or both. Propensity scores that simultaneously adjust for all observed confounders and do not depend on any other covariates - observed or otherwise - can be used to ensure that the observed confounders are similarly distributed (i.e., “balanced”) in the treated and untreated groups [64]. Hence, we will adopt the DR-AIPW estimator in developing the proposed sensitivity analysis for the above reasons.

In this paper, we exploit *covariate balancing propensity scores* (CBPS) [65] to estimate the subgroup-specific propensity score model for treatment given covariates. CBPS estimators of the model coefficients maximize covariate balance toward eliminating confounding bias, whereas conventional maximum likelihood estimators optimize predictive accuracy [66], potentially leading to unstable weights.^3^ In the applied examples used to illustrate the proposed method later, we will adopt a logistic regression model with main effects for the covariates as the propensity score model. Furthermore, for the subgroup-specific outcome model, we will consider a saturated logistic regression model with all possible interactions between the treatment and the covariates. To avoid overfitting in the outcome model, we then utilize *elastic net* regularization or penalization [69] to estimate the coefficients. The elastic net penalty is a mixture of the ridge regression [70] penalty and least absolute shrinkage and selection operator (LASSO) [71] penalty. The ridge regression penalty partially deletes all variables by shrinking the coefficient estimates toward, but not entirely to, zero. In contrast, the LASSO penalty selects variables by setting a coefficient estimate precisely to zero, thus completely deleting that variable. The elastic net inherits the benefits of both penalties and is especially useful when there are many correlated variables [72]. In the current context, the covariates can be indicators of the latent subgroup, and therefore highly correlated due to their shared dependence (on the latent subgroup).

### Implications of misclassification

Frequently in practice, the latent subgroup memberships $\varvec{C}^{*}$ are unknown and have to be imputed. Let $C_{i}$ denote an imputed subgroup for individual *i*, where dependence on a statistical model for obtaining $C_{i}$ is implied and omitted for notational convenience. Denote the resulting vector of imputed subgroup memberships by $\varvec{C}=(C_{1},\ldots ,C_{n})$. The subgroup-specific estimator given a vector of imputed subgroup memberships $\varvec{C}$ is obtained by plugging in $\varvec{C}$ for $\varvec{C}^{*}$ in (2). Given the imputed subgroup memberships $\varvec{C}$, let $\hat{p}(\varvec{X}_{i},C_{i}=c)$ and $\hat{m}(Z_{i}, \varvec{X}_{i}, C_{i}=c)$ denote the estimated individual propensity scores and predicted outcomes, respectively, among those in the partition with $C_{i}=c$, and let $\widehat{\tau }_{c}(\varvec{C})$ denote the resulting effect estimator. But when individuals are misclassified, i.e., $\varvec{C} \ne \varvec{C}^{*}$, so that the imputed subgroups are contaminated by individuals from different latent subgroups, and the (true) propensity score and outcome models differ across different subgroups, then the estimated propensity score model $\hat{p}(\varvec{X}_{i},C_{i}=c)$ and the estimated outcome model $\hat{m}(Z_{i}, \varvec{X}_{i}, C_{i}=c)$ are inconsistent for the true models $p(\varvec{X}_{i},C_{i}^{*}=c)$ and $m(Z_{i}, \varvec{X}_{i}, C_{i}^{*}=c)$, respectively. The subgroup-specific (average causal) effects are thus unidentified under misclassification [13], even when there is no unmeasured confounding of the treatment and the outcome.

## Sensitivity analysis to classification uncertainty

When the imputed subgroup memberships are obtained using finite mixture models, each individual has (typically nonzero) estimated predictive or posterior probabilities of belonging to each distinct subgroup. Denote the estimated probabilities of an individual *i* belonging to each possible subgroup by $\widehat{\lambda }_{ic} \ge 0, c \in \mathcal {C}$, where $\sum \nolimits _{c} \widehat{\lambda }_{ic} = 1$. Similar to $C_{i}$, the dependence on a statistical model for estimating $\widehat{\lambda }_{ic}$ is implied and omitted for notational convenience. Under modal assignment, an individual’s imputed (latent) subgroup is determined by the most likely subgroup they belong to (with the largest probability); i.e., $C_{i} = \text {arg}\,\text {max}_{c \in \mathcal {C}} \widehat{\lambda }_{ic}$. Holding the imputed subgroup memberships fixed when subsequently estimating the subgroup-specific effects ignores information conveyed by the probabilistic memberships. For example, suppose that individual *i* has subgroup membership probability $\widehat{\lambda }_{i1}=0.51$, whereas another individual *j* has probability $\widehat{\lambda }_{j1}=0.98$. While both individuals have the same imputed subgroup when estimating the subgroup-specific causal effect, between the two individuals, individual *i* is more likely to be misclassified. It is thus judicious to assess the change in the subgroup-specific effect estimates if individual *i* did not belong to subgroup 1, ahead of individual *j*. We build on this idea to develop a strategy for assessing the sensitivity of inference about the subgroup-specific effects to classification uncertainty.

### Trajectories of subgroup-specific treatment effects

We propose assessing the impact of possible misclassification on a subgroup-specific effect by methodically considering different partitions of individuals belonging to that subgroup. In particular, we exploit the probabilistic subgroup memberships by ordering the individuals according to their (estimated) values of $\widehat{\lambda }_{ic}$ for a particular subgroup *c*. Let $\widehat{\mathcal {S}}_{c}(m)$ denote the partition of *m* individuals belonging to subgroup *c*; the “hat” symbol denotes the dependence on $(\widehat{\lambda }_{ic}, i=1,\ldots ,n,c \in \mathcal {C})$. Starting with the empty set $\widehat{\mathcal {S}}_{c}(0) = \emptyset$, repeat the following steps for $j = 1,\ldots ,n$ in turn:

1. Let $i^{*}$ index the individual with the largest subgroup membership probability, among all individuals: (i) who are not currently in $\widehat{\mathcal {S}}_{c}(j-1)$, and (ii) whose imputed subgroup would be *c*; i.e.,
  6 $$\begin{aligned} i^{*} = {\underset{i}{\text {arg}\,\text {max}}} \widehat{\lambda }_{ic} \times \mathrm {I}\{i \not \in \widehat{\mathcal {S}}_c(j-1)\} \times \mathrm {I}(c= \underset{c^{\prime} \in \mathcal {C}}{\text {arg}\,\text {max}}\, \widehat{\lambda }_{ic^{\prime }}). \end{aligned}$$
  If $\widehat{\mathcal {S}}_{c}(j-1)$ already includes all individuals whose imputed subgroup would be *c*, so that none of the remaining candidate individuals would have been imputed to the subgroup *c* under modal assignment, then determine $i^{*}$ simply as (the index of) the individual with the largest subgroup membership probability; i.e.,
  7 $$\begin{aligned} i^{*} = {\underset{i}{\text {arg}\,\text {max}}} \widehat{\lambda }_{ic} \times \mathrm {I}\{i \not \in \widehat{\mathcal {S}}_{c}(j-1)\}. \end{aligned}$$
2. Add individual $i^{*}$ to $\widehat{\mathcal {S}}_{c}(j-1)$ to determine the next partition; i.e., $\widehat{\mathcal {S}}_{c}(j) = i^{*} \bigcup \widehat{\mathcal {S}}_{c}(j-1)$.
3. Calculate the subgroup-specific (average treatment) effect estimate among individuals indexed by $\widehat{\mathcal {S}}_{c}(j)$.

The largest partition is simply the observed sample $\widehat{\mathcal {S}}_{c}(n) = (1,\ldots ,n)$. The sequence of nested partitions of individuals $\{\widehat{\mathcal {S}}_{c}(j): j=1,\ldots ,n\}$, therefore, induces a sequence of subgroup-specific effect estimates based on an increasing number of individuals (one at a time) belonging to that subgroup *c*. When there exists a partition $\widehat{\mathcal {S}}_{c}(j^{*})$ for some value of $j^{*} \in \{1,\ldots ,n\}$ that includes only individuals actually belonging to subgroup *c* (thereby excluding all individuals who do not belong to subgroup *c*), then the subgroup-specific effect estimate using this partition – consisting only of correctly classified individuals – will be consistent for its population value. In the next section, we will demonstrate, using selected examples from the illustrations, graphical summaries of the subgroup membership probabilities, and the constructed sequence of effect estimates that can be visually inspected to assess the relative stability or (in)sensitivity of the subgroup-specific effect.

We have elected to add individuals who would have been imputed to subgroup *c* ahead of others who would have been imputed to a different subgroup. When there are more than two subgroups, an individual *i* with an imputed subgroup $C_{i}=c$ may nonetheless have subgroup membership probability $\widehat{\lambda }_{ic}$ smaller than another individual *j* with a different imputed subgroup $C_{j} \ne c$. Consider the following simple example with three subgroups. The (posterior) subgroup membership probabilities for two individuals *i* and *j* are respectively: $(\widehat{\lambda }_{i1},\widehat{\lambda }_{i2},\widehat{\lambda }_{i3})=(0.4,0.3,0.3)$ and $(\widehat{\lambda }_{j1},\widehat{\lambda }_{j2},\widehat{\lambda }_{j3})=(0.45,0.55,0)$. Because our focus is on the sensitivity of the effect estimate based on the imputed subgroup memberships (under model assignment), we would add to subgroup 1 individual *i* (who has stronger evidence of belonging to subgroup 1 among all subgroups) ahead of individual *j* (who has weaker evidence relative to subgroup 2). It follows that the effect estimate $\widehat{\tau }_{c}(\varvec{C})$, where the imputed subgroup membership vector $\varvec{C}$ is determined under model assignment using $(\widehat{\lambda }_{ic}, i=1,\ldots ,n,c \in \mathcal {C})$, corresponds to the effect estimate based on $\widehat{\mathcal {S}}_{c}(m_{c})$, where $m_{c} = \sum \nolimits _{i=1}^{n} \mathrm {I}(C_{i}=c)$ denotes the number of individuals imputed to subgroup *c*.

The subgroup-specific effect estimator can be calculated only when the individual weights within that subgroup are well-defined. In particular, the theoretical or deterministic “positivity” assumption states that all true (but unknown) propensity scores must be strictly between zero and one for all values in the covariate space [67, 73]. But the positivity assumption can nonetheless be violated practically or randomly in a finite sample. For example, there may be only treated or untreated individuals, but not both, in the same subgroup, or only specific covariate values are observed in the data at hand so that there is “complete separation” in the fitted propensity score model in a subgroup. Then propensity scores cannot be estimated in each subgroup, and the effect estimate cannot be calculated. Moreover, when there are only a few individuals in a subgroup, the estimated propensity scores can be very close to zero or one, and the resulting weights can take on very large values, leading to highly unstable weights and effect estimates that fluctuate dramatically as individuals are added to (or removed from) that subgroup. Such potentially uninformative fluctuations can be avoided simply by focusing on partitions $$\widehat{\mathcal {S}}_{c}(j)$$ indexed by particular values of *j*. For example, suppose interest is only in individuals whose membership probabilities lie between 0.99 and 0.01. Then let $$m_{c}^{*} = \sum \nolimits _{i=1}^{n} \mathrm {I}(\widehat{\lambda }_{ic} \ge 0.99)$$ be the number of individuals whose probabilities of belonging to subgroup *c* are at least as large as 0.99, and let $$m_{c}^{**} = \sum \nolimits _{i=1}^{n} \mathrm {I}(\widehat{\lambda }_{ic} \ge 0.01)$$ be the number of individuals whose probabilities of belonging to subgroup *c* are at least as large as 0.01. The sequence of subgroup-specific effect estimates could then be calculated based on only the partitions $$\{\widehat{\mathcal {S}}_{c}(j): j=m_{c}^{*}, \ldots , m_{c}, \ldots , m_{c}^{**}\}$$. The user-specified thresholds may be decided based on whether there are insights to be gained from adding individuals (one at a time) whose membership probabilities are outside reasonable thresholds. Because our focus is on the impact of classification uncertainty, we have elected to impose thresholds based on individuals’ predictive or posterior probability or likelihood of belonging to a particular subgroup, rather than (arbitrary) absolute subgroup proportions or sizes alone.

### Graphical assessment of sensitivity to classification uncertainty

In this section, we describe how to visually inspect the relative stability or (in)sensitivity of the constructed trajectories of the subgroup-specific effect estimates from the preceding section. We employ two graphical summaries for each subgroup.

#### Membership probability plot

**Fig. 2:**
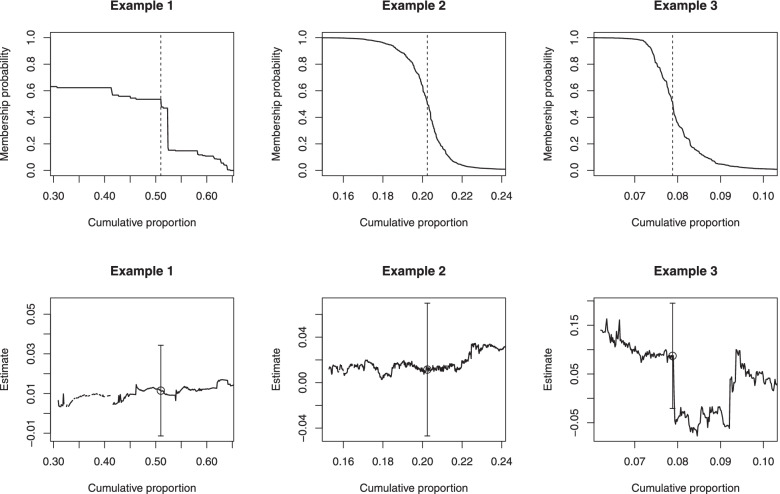
Examples of plots for gauging the (in)stability of the subgroup-specific (average) treatment effect estimates. The plots are for three different subgroups from different datasets. In the top panel, the cumulative proportion of individuals (horizontal axis) whose subgroup membership probabilities are above a certain value (on the vertical axis) are plotted. The vertical broken line indicates the proportion of individuals imputed to that subgroup. In the bottom panel, the treatment effect estimates as individuals are added one at a time to that subgroup are plotted. The empty circles, and error bars, indicate subgroup-specific effect estimates, and 95% CIs, respectively, based on the imputed subgroup memberships

In the first graphical summary for each subgroup, we plot the membership probabilities of individuals belonging to that subgroup, such as those in the top panels of Fig. 2 (for three different examples from different datasets). In each panel, the curve represents the cumulative proportion of individuals (horizontal axis) whose subgroup membership probabilities $\widehat{\lambda }_{ic}$ for that subgroup are greater than or equal to a specified value (on the vertical axis). Therefore, the curve is monotonically decreasing by construction because for any two membership probability values *l* and $l^{\prime }$ where $l < l^{\prime }$, there are at least as many individuals with membership probabilities greater than or equal to *l* as there are for $l^{\prime }$. Each curve graphically depicts the distribution of the membership probabilities across the sample. A curve with a steeper gradient (thus more closely resembling a step function), e.g., Example 1 in the top left panel of Fig. 2, indicates a more distinctive partition of individuals who either belong (with membership probabilities closer to one) or do not belong (with membership probabilities closer to zero), to that subgroup. In contrast, a curve with a gentler gradient, e.g., Examples 2 and 3 in the top center and right panels of Fig. 2, suggests that there are “tentative” individuals whose probabilities of belonging to that subgroup may be closer to 0.5. The presence of (many) tentative individuals suggests an unclear separation of individuals into members and non-members of that latent subgroup. We reiterate that our interest is not merely in the values of the probabilities but in how well-separated possible members and non-members in each latent subgroup are. Finally, a vertical broken line demarcates the proportion of individuals imputed to a subgroup.

#### Treatment effect trajectory plot

In the second graphical summary for each subgroup, we plot the trajectory of the subgroup-specific average treatment effect estimates described in the preceding section. The trajectory is indexed by the sequence of (nested) partitions of individuals added one at a time to that subgroup, such as those shown in the bottom panels of Fig. 2. To aid visual clarity, we will simply plot (on the horizontal axis) the same cumulative proportion of individuals belonging to each subgroup as for the membership probabilities in the corresponding panel above. The values of the effect point estimates are plotted on the vertical axis. (The vertical axes in Fig. 2 differed because these examples are drawn from different datasets.) We recommend using the same scale and range of values displayed across the classes for a single dataset, as we will demonstrate using the applied examples in the next section. An empty circle in each panel marks the subgroup-specific effect estimate based on the imputed subgroup memberships. Its value on the horizontal axis is the same as that of the vertical broken line in the corresponding panel above. In these examples, we imposed thresholds of 0.99 and 0.01 for the membership probabilities to focus attention on the (in)stability of the trajectory due to individuals who could reasonably be considered as potentially belonging to that subgroup.

We can then gauge the relative stability of the subgroup-specific effect estimates when either fewer or more (tentative) individuals are presumed to belong to that subgroup compared to those imputed to that subgroup. The uncertainty in the estimates can be (partially) accounted for by assessing its stability relative to the 95% CI based on the imputed subgroup memberships, which are plotted simply as vertical error bars. In Examples 1 and 2 (bottom left and middle panels of Fig. 2), the subgroup-specific effect estimates were relatively stable even as fewer (or more) tentative individuals were in that subgroup, with the point estimates remaining within the 95% CI. In contrast, in Example 3 (bottom right panel of Fig. 2), the subgroup-specific effect estimates displayed larger fluctuations. In particular, when the subgroup comprised more individuals (with smaller membership probabilities), the estimates were outside the lower bound of the CI that was based on the imputed memberships. Such instability suggests a sensitivity to potential misclassification beyond the sampling uncertainty captured by the 95% CI, which merely assumes the imputed subgroups to be fixed. In the next section, we describe how to construct perturbed CIs that account for sampling uncertainty in both the classification and effect estimation models. An unstable trajectory with values that are either outside the CI or (dramatically) different for different subsets of individuals suggests a possibility of contamination by members of other subgroups. Fitting a local smoother, or calculating moving averages, can be used to further inspect the (in)stability of the trajectory numerically; we defer exploring such methods to future work. Caution when interpreting the subgroup-specific effects is thus advised, and investigators should revisit the definitions of the latent subgroups and models for estimating the membership probabilities.

### Perturbed confidence intervals

Because each individual can have non-zero probabilities of belonging to different classes, multiple pseudo-class draws of the imputed class memberships, e.g., assuming a binomial or multinomial distribution with the given probabilities can be made to classify individuals stochastically [74]. But such an approach accounts for only the imprecision from coarsening the probabilistic class memberships to deterministic partitions and is no better than a single modal assignment [43]. Whereas the imputed subgroup memberships are (prediction) error-prone measures of the latent subgroup memberships, the estimated subgroup membership probabilities, based on a membership model fitted to the observed sample, are subject to sampling uncertainty. Continuing the example above, suppose that the individual with subgroup membership probabilities $(\widehat{\lambda }_{j1},\widehat{\lambda }_{j2},\widehat{\lambda }_{j3})=(0.45,0.55,0)$ actually belonged to subgroup 1, but the estimated values of $\widehat{\lambda }_{j1}<\widehat{\lambda }_{j2}$ were due to sampling variability in the estimated parameters of the finite mixture model. Our interest is not merely imputing this individual to subgroup 1 or 2, either 45% or 55% of the time over repeated random classifications, but in acknowledging the sampling uncertainty in the probabilities themselves.

To more honestly reflect the uncertainty in the estimated subgroup membership probabilities when carrying out inference of the subgroup effects, we propose *perturbing* the probabilities as follows:

1. Randomly draw a value of the subgroup membership model parameter estimates from their joint sampling distribution, which we denote simply by $\hat{G}(\cdot )$. For each individual $i=1,\ldots ,n$, calculate the perturbed subgroup membership probabilities, which we denote by $(\tilde{\lambda }_{ic}, c \in \mathcal {C})$, using the $\sim$ symbol, after plugging in the randomly drawn values of the parameters in the subgroup membership model. Details on how to carry out this step under each of two common finite mixture modeling approaches, including examples using freely available R packages, are provided in the Online Supplemental Materials.
2. Given the perturbed subgroup membership probabilities $(\tilde{\lambda }_{ic}, i=1,\ldots ,n,c \in \mathcal {C})$, determine each individual’s (perturbed) imputed subgroup using modal assignment as $\tilde{C}_{i} = \text {arg}\,\text {max}_{c \in \mathcal {C}} \tilde{\lambda }_{ic}$. Calculate each subgroup-specific effect with the resulting perturbed subgroup memberships $\tilde{\varvec{C}}=(\tilde{C}_{1}, \ldots , \tilde{C}_{n})$. For each perturbation, construct the $100(1-\alpha )\%$ CI under the (perturbed) imputed subgroup memberships $\tilde{\varvec{C}}$.
3. Repeat both steps above, e.g., 1000 times. Combine the individual CIs across all perturbations to determine a “perturbed” CI by setting the lower (or upper) endpoint to be the 2.5 (or 97.5) percentile among the lower (or upper) endpoints of all the individual CIs.

To reduce the risks of label switching in practice, the subgroups in each perturbation can be relabelled to maximize the similarity between the modal assignment based on the perturbed probabilities and the estimated class memberships. We elected to eliminate extreme endpoints for CIs from individual perturbations to improve the insensitivity of the resulting (combined) perturbed CI to extreme values. In principle, the perturbed CI may be constructed using the union method instead, where the lower (upper) endpoint is the minimum (maximum) among all the separate lower (upper) endpoints for the CIs. However, such intervals are susceptible to a single perturbation that yields extreme CIs and may potentially be unduly conservative with coverage levels exceeding their nominal levels [75].

In the Online Supplemental Materials, we use simulated examples to empirically demonstrate that merely holding the imputed subgroup memberships fixed can lead to CIs that contain the true subgroup effect (far) below the nominal coverage level. In contrast, the perturbed CIs include the true subgroup effect more frequently, albeit possibly below the nominal coverage level, because the bias remains uncorrected. Moreover, we evaluate empirically via Monte Carlo simulation the ability of a constructed trajectory of the subgroup-specific effect estimates – using only the estimated subgroup membership probabilities – to recover the true effect. We consider subgroups defined as either latent classes in a latent class model or mixture components in a Gaussian mixture model.

In general, pointwise confidence bands for the trajectory of each subgroup-specific effect can be similarly constructed using the perturbed probabilities; we defer details to the Online Supplemental Materials. Such a parametric bootstrap approach has been employed in other contexts, e.g., item response theory score estimation [76, 77]. Finally, while standard errors of coefficient estimates in parametric models that account for the uncertainty in the estimated class memberships are available [78], such parametric approaches are limited to outcome models that parametrize the treatment effect as a regression coefficient. Extending such approaches to the DR-AIPW estimator utilized in this paper is a direction for future work.

## Applied examples using real-world data

### Percutaneous Coronary Intervention

The “lindner” dataset was from an observational study on the effectiveness of an augmented Percutaneous Coronary Intervention (PCI) on six-month survival. The dataset is publicly available as part of the PSAgraphics [79] and twang [80] packages in R, and contains information on 996 patients at the Lindner Center, Ohio Heart Health, Cincinnati in 1997. We utilized the version of the dataset available as part of the twang package. The treatment was whether a patient received usual PCI treatment alone ($Z=0$) or PCI treatment deliberately augmented by a cascade blocker Abciximab ($Z=1$); 698 patients received Abciximab. The outcome was whether a patient survived to six months ($Y=1$) or not ($Y=0$); 970 patients survived to six months. The following covariates were recorded: whether the patient suffered from a recent acute myocardial infarction within the previous seven days or not; their left ventricle ejection fraction (percentage between 0 and 90); the number of vessels involved in an initial PCI procedure (integer between 0 to 5); whether a coronary stent was deployed or not; whether the patient had been diagnosed with diabetes mellitus or not; the patient’s height in centimeters; and whether the patient was female or male.

For the sole purpose of illustration, we considered a latent class model with the five covariates measuring the patients’ medical and health history as manifest indicators of the latent class. Among the manifest indicators, only the ventricle ejection fraction variable was continuous; the remaining variables were categorical. Hence, solely for fitting a latent class model to the observed covariates in this illustrating example, the continuous variable was discretized by binning into the sample quintiles to obtain a (coarsely discrete) categorical variable. We fitted different candidate measurement models for the latent class and its indicators only, each with a fixed number of latent classes between two and ten (so that there were nine candidate models in total), using the poLCA package [81] in R. We selected the model with two latent classes because it minimized the BIC, and the AIC was only slightly larger than the minimum value for the three-class model. We report the average value for each of the five manifest indicators by latent class in Table 1. These values indicated the probability (if the indicator was binary) or average quintile (if the indicator was discretized) that a patient representative of that class would exhibit for that characteristic.

**Table 1:** Average value for each manifest indicator used in the measurement model by latent class for the lindner data. The *p*-value from a Chi-squared test of the frequency table of class membership versus values of each indicator is displayed in the rightmost column. The estimated proportion in each class is stated in the last row. All results were rounded to three decimal places

Indicator	Class 1	Class 2	*p*-value
Coronary stent (stent)	0.717	0.639	0.003
Diagnosed with diabetes mellitus (diabetic)	0.196	0.241	0.006
Recent acute myocardial infarction (acutemi)	0.000	0.230	0.000
Left ventricle ejection fraction (ejecfrac)	2.643	1.077	0.000
Number of vessels involved in an initial PCI procedure (ves1proc)	1.324	1.423	0.000

Because treatment was non-randomly assigned (patients who received Abciximab tended to be more severely diseased and thus more likely to suffer from six-month mortality), the PS and outcome models with all available (discretized) covariates were fitted within each imputed class to calculate the DR-AIPW effect estimator. Both models included the two demographic variables (height and gender) excluded from the latent class model. We assumed no unmeasured confounding within each class after adjusting for all the measured covariates. The class-specific effect estimates (listed in increasing order of the lower endpoint of the 95% CI shown in parentheses) based on the imputed class memberships are displayed in the second row of Table 2. The results suggested a positive effect of augmented PCI only among about half the patients. These were patients who were more likely to have had a recent acute myocardial infarction within the previous seven days but with a smaller number of vessels involved in an initial PCI procedure. Nonetheless, there was insufficient evidence of treatment effect heterogeneity between the estimated classes (due to the overlapping CIs). In contrast, the DR-AIPW estimator for the entire sample was 0.06 with a 95% CI of (0.02, 0.10), suggesting a beneficial average effect across all individuals.

**Table 2:** Estimated class proportions (top row), and class-specific average treatment effects (second and third rows), for the latent classes in the lindner data. The class-specific confidence intervals (CIs) were based on estimated class memberships that were either held fixed (second row), or based on perturbed probabilities that accounted for the uncertainty in the estimates (third row). The classes were listed in increasing order of the lower endpoints of the CIs based on the estimated memberships. All results were rounded to two decimal places

Latent class	1	2
Proportion	0.51	0.49
Fixed	0.01 (-0.01, 0.03)	0.09 (0.03, 0.16)
Perturbed	0.01 (-0.02, 0.05)	0.09 (0.02, 0.30)

**Fig. 3:**
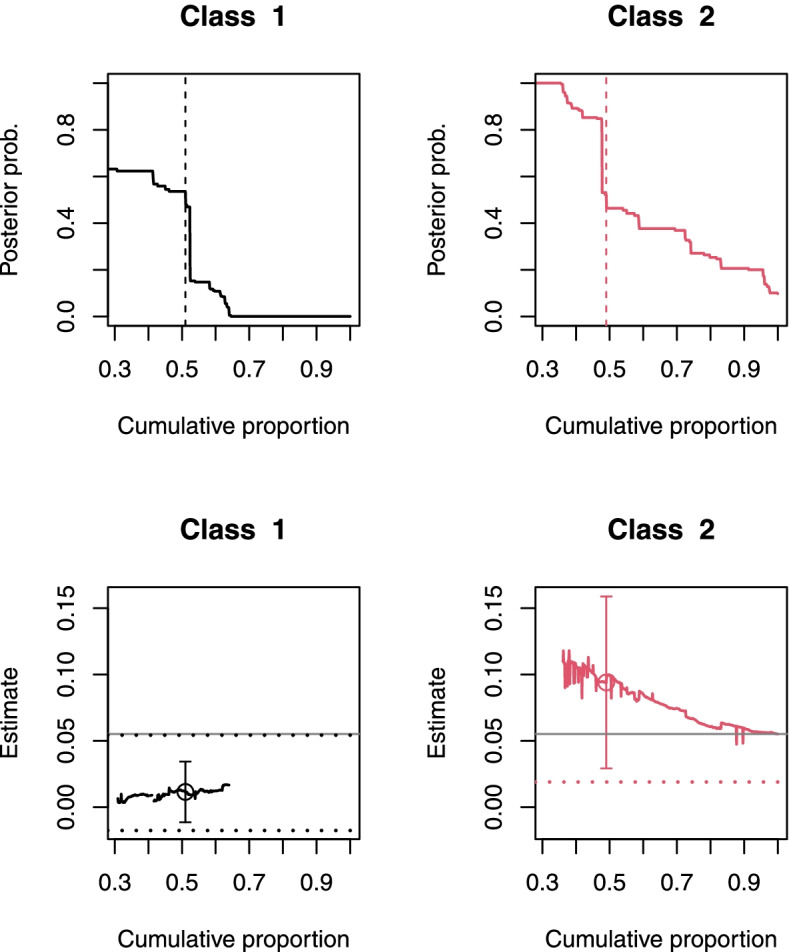
Plots for assessing the stability of the class-specific (average) treatment effect estimates in the lindner data. Details on how to interpret each plot are described in Section 3.2 and in the caption of Fig. 2. The endpoints of the perturbed CI for the effect within each class are plotted as horizontal dotted lines. The upper endpoint of the CI for class 2 is much larger than that of the other class and thus omitted to improve visualization. The sample average treatment effect is plotted as a horizontal solid gray line

The graphical summaries of the posterior membership probabilities of individuals belonging to each latent class, and the trajectories of the class-specific effect estimates as individuals were added one at a time to each class, are plotted in the top and bottom panels of Fig. 3 respectively. Neither latent class was perfectly separated, as indicated by the gradients of the curves in the top panels, with tentative individuals in each class. The trajectories displayed in the bottom row of panels suggested that the class-specific effect estimates for both classes appeared stable relative to the 95% CI based on the imputed class memberships. The positive treatment effect in class 2 gradually tended toward zero only as more individuals, possibly from class 1, were added to that class. The endpoints of the perturbed 95% CIs are displayed in the third row of Table 2 and plotted as horizontal dotted lines in the bottom row of Fig. 3. While the perturbed CI for class 1 was slightly wider than that based on the imputed memberships, the perturbed CI for class 2 was much wider. But the average effect of augmented PCI on six-month survival among individuals in class 2, which constituted about half the sample, remained statistically significant (at 5% level), even after accounting for the sampling variability in the estimated membership probabilities, with a perturbed 95% CI of (0.02, 0.30).

### Right Heart Catheterization

The “RHC” dataset was from an observational study on the effectiveness of Right Heart Catheterization in the initial care of critically ill patients [11]. It was distributed as part of the Hmisc package in R. The dataset contained information on hospitalized adult patients at five medical centers in the U.S. who participated in the Study to Understand Prognoses and Preferences for Outcomes and Risks of Treatments (SUPPORT). The treatment was whether a patient received an RHC within 24 hours of admission ($Z=1$) or not ($Z=0$); 2184 patients received an RHC. The outcome was whether a patient died at any time up to 180 days since admission ($Y=1$) or not ($Y=0$); 3722 patients died within the considered timeframe. There were 5735 participants with 73 covariates.

For the sole purpose of illustration, we considered a latent class model with 60 of the covariates measuring the patients’ medical and health history as manifest indicators of the latent class. Among the manifest indicators, 20 were continuous; each was discretized by binning into the sample quintiles to obtain (coarsely discrete) categorical variables. We fitted six possible (measurement) models for the latent class and its indicators only, each with a different number of latent classes (between two and seven^4^), using the poLCA package [81] in R. We selected the model with four latent classes because it minimized the AIC and BIC, and the subgroup-specific DR-AIPW estimators given the imputed class memberships could be calculated. While larger models (five to seven-class models) had lower values of the AIC and BIC, the subgroup-specific effect estimator could not be calculated for certain imputed classes that either contained only treated or untreated or had only survived or deceased individuals. Due to space constraints, we report the average value for each of the manifest indicators in the Online Supplemental Materials.

Because treatment was non-randomly assigned (patients who received an RHC tended to have existing health or medical conditions, and thus more likely to suffer from six-month mortality), the PS and outcome models with all available (discretized) covariates were fitted within each imputed class to calculate the DR-AIPW effect estimator. Both models included the 13 demographic and socioeconomic status variables (age, gender, ethnicity, years of education, income, and health insurance) excluded from the latent class model. We assumed no unmeasured confounding within each class after adjusting for all the measured covariates. The class-specific effect estimates (listed in increasing order of the lower endpoint of the 95% CI shown in parentheses) based on the imputed class memberships are displayed in the second row of Table 3. The results suggested a harmful effect of RHC only among about 40% of the patients. These were patients who were more likely to have had multiple organ system failure (MOSF) with sepsis, cirrhosis, gastrointestinal diagnoses, chronic renal disease or hemodialysis, or upper GI bleeding and were less likely to have cancer. Nonetheless, there was insufficient evidence of treatment effect heterogeneity between the estimated classes (due to the overlapping CIs). In contrast, the DR-AIPW estimator for the entire sample was 0.06 with a 95% CI of (0.04, 0.09), suggesting a statistically significant harmful average effect across all individuals.

**Table 3:** Estimated class proportions (top row), and class-specific average treatment effects (second and third rows), for the latent classes in the RHC data. The class-specific confidence intervals were based on estimated class memberships that were either held fixed (second row), or based on perturbed probabilities that accounted for the uncertainty in the estimates (third row). The classes were listed in increasing order of the lower endpoints of the CIs based on the estimated memberships. All results were rounded to two decimal places

Latent class	1	2	3	4
Proportion	0.20	0.32	0.08	0.39
Fixed	0.01 (-0.05, 0.07)	0.04 (-0.03, 0.11)	0.09 (-0.02, 0.20)	0.08 (0.03, 0.13)
Perturbed	0.01 (-0.05, 0.07)	0.04 (-0.04, 0.12)	0.09 (-0.57, 0.31)	0.08 (0.03, 0.14)

**Fig. 4:**
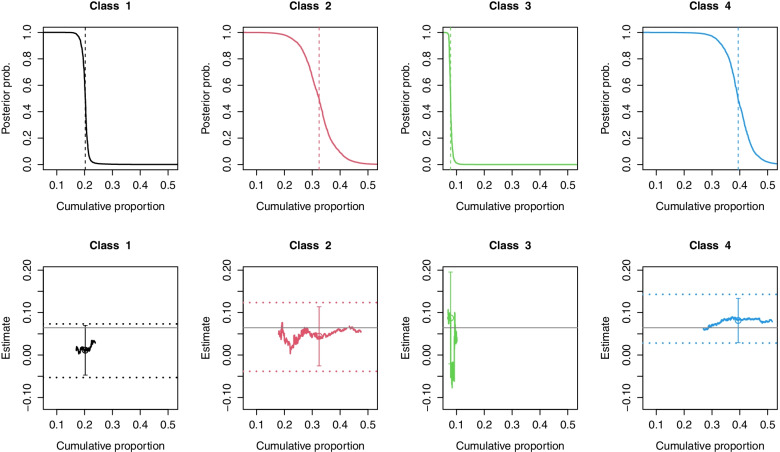
Plots for assessing the stability of the class-specific (average) treatment effect estimates in the RHC data. Details on how to interpret each plot are described in Section 3.2 and in the caption of Fig. 2. The endpoints of the perturbed CI for the effect within each class are plotted as horizontal dotted lines. The CI for class 3 is much wider than those of the other classes and thus omitted to improve visualization. The sample average treatment effect is plotted as a horizontal solid gray line

The graphical summaries of the posterior membership probabilities of individuals belonging to each latent class, and the trajectories of the class-specific effect estimates as individuals were added one at a time to each class, are plotted in the top and bottom rows of panels of Fig. 4 respectively. None of the four latent classes were perfectly separated, as indicated by the gradual gradients of the curves in the top panels, with tentative individuals in each class. The trajectories displayed in the bottom row of panels suggested that the class-specific effect estimates were relatively stable for classes 1, 2, and 4, making up about 92% of the sample. However, the trajectory of estimates for the remaining 8% of patients in class 3 fluctuated as more individuals were added to that class, beyond the sampling variability under the imputed class memberships. The endpoints of the perturbed 95% CIs are displayed in the third row of Table 3 and plotted as horizontal dotted lines in the bottom row of Fig. 4. While the perturbed CIs for classes 1, 2, and 4 were slightly wider than those based on the imputed memberships, the perturbed CI for class 3 was much wider. The average effect of RHC on six-month mortality among individuals in class 4, which constituted about 40% of the sample, remained statistically significant (at 5% level), even after accounting for the sampling variability in the estimated membership probabilities, with a perturbed 95% CI of (0.03, 0.14).

## Discussion

### Comparisons with existing work using latent class models

When latent class models are used to impute the individual subgroup memberships, specific existing bias correction procedures may be considered. Bray et al. [12] recommend an “inclusive latent class analysis” where the individual posterior probabilities of belonging to each latent class, and the estimated average potential outcomes, are conditioned on the same set of covariates. In the latent class model (in the first “classify” step), all observed baseline covariates that are conditioned on in the class-specific effect estimator (in the second “analyze” step) should thus be included as independent “concomitant” predictors or explanatory variables. However, as demonstrated in the applied examples, assuming such “concomitant-variable” latent class models [82] may require distinguishing between observed covariates that are either manifest (“auxiliary” response) indicators, or concomitant (independent) predictors, of the latent classes [83]. Gardner [13], following [41] and [42], derives unbiased estimators of the true latent class-specific average potential outcomes that correct for potential errors in the imputed latent class memberships. However, the validity of the estimator is predicated on assuming conditional independence between the latent class indicators and external observed variables given the latent class. Recent modifications of bias-adjusted methods that allow for violations of this assumption are limited to settings with “one or two” ([45], p.361) external variables, which can be unrealistic when there are several covariates (i.e., explanatory variables) that directly affect the indicators, which in turn directly affect both treatment and outcome (i.e., “distal” outcomes). Moreover, it is unclear whether non-continuous external variables are permitted and whether these methods are implemented in R outside of specific software [84, 85].

In this paper, we have focused on how the effects of an observed treatment on an observed outcome may differ among individuals belonging to different latent classes defined using the observed covariates. Lanza et al. [86] and Clouth et al. [87] consider settings where an observed treatment affects an unobserved outcome, whose levels take the form of latent classes while adjusting for observed covariates that serve as explanatory variables for the latent classes. In contrast, Bray et al. [12], Bray et al. [88], and Schuler [89], consider settings where an unobserved treatment, whose levels take the form of latent classes, affect observed outcomes while adjusting for observed covariates that serve as explanatory variables for the latent classes. We have focused on settings where, in the context of latent class analysis, the observed treatment and outcome are both “distal outcome” (response) variables that are affected by the latent (explanatory) class. In principle, latent class methods for estimating the association between latent (explanatory) classes and observed (response) variables when combining measurement models with structural regression models [42, 90], may be used to estimate class-specific treatment effects. But these methods would demand additional stringent assumptions about (i) how the latent class variable moderates the effect of treatment on the outcome, and (ii) adjusting for the observed confounders of treatment and outcome in the (correctly-specified linear) outcome model; see, e.g., [91] and [92]. Mayer et al. [92] consider effect heterogeneity by assuming covariate-treatment (statistical) interaction terms to evaluate (conditional) treatment effects for all unique combinations of the (observed) covariates. Moreover, outcome regression models which demand specifying complex interactions between possible moderators with treatment, such as (latent) class-treatment, covariate-treatment, and class-covariate-treatment terms, can become unwieldy, unstable, and uninterpretable when there are more than a handful of covariates. In contrast, we propose using an estimator that (i) permits either continuous or non-continuous outcomes by accommodating non-linear models for the latter, and (ii) is not contingent on correctly modeling the outcome in terms of (possibly complex) functions of the latent classes, treatment, and covariates.

### Limitations and future directions

There are several avenues for future research that extend the ideas developed in this paper. When substantive interest is in assessing treatment effects on the outcome that are moderated by (potentially complicated functions of) the covariates [92], a finite mixture regression model [37] for the outcome may be considered as a more parsimonious parametric alternative for categorizing individuals. More parsimonious approaches to fitting the estimation models within each class have the benefits of borrowing information on the confounding mechanisms across classes and easier model interpretation. But such approaches would require extending the DR-AIPW estimator to allow for such an outcome model; moreover, perturbing the class membership probabilities would require accounting for the (joint) sampling variability of the parameters in the outcome model and the propensity score model. Moreover, it is rarely known in practice the extent to which confounding mechanisms are either similar or differ across classes. A flexible approach, therefore, permits researchers to utilize any suitable estimation method within each class. In future work, we will explore effect estimators using data-adaptive nonparametric machine learning-based algorithms which utilize sample splitting, cross-fitting, and averaging to reduce the risk of overfitting [93], and compare them with parametric approaches which permit correcting for classification errors but may be prone to structural model misspecification.

For the sole purpose of illustration, we made three simplifying assumptions in the applied examples. First, because common latent class methods are restricted to manifest indicators being (unordered) categorical variables, we discretized continuous variables which were indicators of the latent classes. In general, indicators can consist of categorical and continuous variables. Hence, an alternative to latent class analysis under such settings is to implement model-based clustering of a combination of binary and Gaussian data, such as FLXMCmvcombi in the flexmix package [94]. Second, as with other latent class methods, we assumed the number of latent classes to be known a priori, and we used the widely adopted AIC and BIC model selection criteria to determine the number of classes. Hence, as with other latent class methods, a specified subgroup or class membership model can only be fitted to observed data with a sufficiently large sample size and a relatively small number of latent classes. A further complication for researchers seeking to test class-specific treatment effects is that they must further take into account the feasibility of fitting fully class-specific structural models. Third, we assumed that the missing data are missing completely at random, so that a complete case analysis is appropriate [95]. In practice, researchers should consider other methods which allow for missing data when estimating latent class models with concomitant variables or multilevel latent class models, such as MultiLCIRT [96]. For example, [97] proposed a causal latent class model using a dynamic propensity score method to estimate weights in order to discover latent subgroups of patients with a latent class model estimated with the MultiLCIRT package.

In this paper, we have defined subgroups using the baseline covariates alone. Alternatively, the classes may be defined as part of a propensity score model that simultaneously fits more than one regression to the observed data with unknown partitions. For example, [25] implemented such finite mixture logistic regression models as propensity score models (under the multilevel setting) that partition the treatment selection processes into distinct latent classes. But a finite mixture logistic regression (with more than one component) for a binary dependent variable is unfeasible in general because a Bernoulli mixture model (with only one trial) is identifiable if and only if there is just one component [98, 99]. Extending the proposed sensitivity analysis to multilevel mixture logistic regression models for the propensity score would thus require different approaches that preserve the cluster structure when adding individuals to each subgroup. The proposed method can be readily adapted for quantitative or continuous treatments by utilizing generalized propensity scores for the treatment [100]. For example, discrete mixtures of linear regression models [94] can be utilized to estimate the subgroup-specific generalized propensity score models. Extending the proposed sensitivity analysis to longitudinal data, such as when treatment directly influences the probability of being in a certain latent class on the first time occasion and the probability to transit from a certain latent class to another over time [101], is complicated. Because the latent class membership probabilities change over time, the constructed partitions – and subsequently trajectories of class-specific effect estimates – may similarly change over time, which can make assessing stability challenging.

Permitting unmeasured confounding due to misclassification (e.g., when unmeasured confounding is limited to a particular subgroup [102], and individuals from that subgroup are misclassified) introduces an additional source of bias. Conversely, misclassification may potentially result from unmeasured confounding when variables that are simultaneously indicators of the latent subgroups and common causes of treatment and outcome are unmeasured. For simplicity, we have focused on the conceptual development of the sensitivity analysis procedure assuming all confounders are measured and defer addressing more complex scenarios with potentially different sources of biases to future work. Finally, we considered just two different classes of finite mixture models where the covariates were used exclusively to measure the latent classes merely to motivate the proposed sensitivity analysis procedure. In principle, nonparametric soft clustering approaches, such as “possibilistic fuzzy C-means” [103] may be accommodated in the proposed strategy. Such methods partition individuals into distinct classes without assuming a latent (parametric) model for the observed data distribution, allowing each individual to belong to multiple classes simultaneously. Membership grades used to measure the degree to which each individual belongs to each given class may be derived to sum to one across the classes, but need not represent a probabilistic measure for ordering the individuals by their (decreasing) likelihood of misclassification. Further work is also required to quantify the uncertainty in the individual membership grades toward perturbing the class membership probabilities when assessing the trajectories of class-specific effect estimates.

## Supplementary Information

**Additional file 1:** Supplementary Material.

## Data Availability

All R scripts used in the illustrations and the simulation studies are freely available on GitHub at https://github.com/wwloh/heterogeneous-effects-under-misclassification. The datasets supporting the conclusions of this article are available as part of the twang (https://CRAN.R-project.org/package=twang) and Hmisc (https://CRAN.R-project.org/package=Hmisc) packages on The Comprehensive R Archive Network.

## Footnotes

1 We acknowledge that in principle when each individual’s predicted or posterior probability of belonging to a specific subgroup is 100%, misclassification can essentially be ruled out under the maximum a posteriori probability allocation rule. In such an ideal but practically uncommon scenario, the proposed method in this paper would then be unnecessary.

2 https://github.com/wwloh/heterogeneous-effects-under-misclassification

3 In practice, the weights can be trimmed by progressively truncating extreme values in the tails to interior percentiles of the initial estimated propensity score distribution [67]. Evaluating specific methods for trimming propensity scores [68] is beyond the scope of this paper.

4 The algorithm for an eight-class model failed to converge.
