# Supplementary material for: Evaluating sensitivity to classification uncertainty in latent subgroup effect analyses
Source: BMC Med Res Methodol. 2022 Sep 24;22:247. doi: 10.1186/s12874-022-01720-8 (PMC9508766; doi:10.1186/s12874-022-01720-8)
Supplement: Supplementary file 1 — Additional file 1: Supplementary Material. [file 12874_2022_1720_MOESM1_ESM.pdf]

Online Supplemental Materials for 'Evaluating Sensitivity to Classification Uncertainty in Latent Subgroup Effect Analyses'

Wen Wei Loh[1] and Jee-Seon Kim[2]

[1]Department of Data Analysis, Ghent University, Gent, Belgium.

[2]Department of Educational Psychology, University of Wisconsin-Madison, USA

A Calculating the perturbed subgroup membership probabilities under finite mixture models

We now describe how to calculate the perturbed subgroup membership probabilities using two common finite mixture models to define and estimate the latent subgroups. We state the specific steps using freely available R packages to enhance accessibility and encourage reproducibility.

A.1 Latent class analysis

When the classes are measured by multiple categorical variables, a latent class analysis is commonly employed to characterize partitions of individuals with similar patterns of the manifest variables. There are two sets of probabilities parametrizing the latent class model:

one describes class-specific cell probabilities in multi-way contingency tables enumerating all possible combinations of individual responses to the categorical variables, and another describes ("prior" mixing) probabilities representing the population distribution of the classes. To obtain the perturbed class membership probabilities, we propose sampling the parameters using a *parametric bootstrap* approach [Dias and Vermunt, 2006] as follows. We describe the steps using the poLCA package [Linzer et al., 2011] for fitting latent class models.

a. Fit the latent class model to the observed data.

```
# X1, X2, X3: manifest variables
# obs_data: observed dataset
mixOut <- poLCA(cbind(X1, X2, X3)~1, data=obs_data, nclass=2)
```

b. Simulate a dataset under the fitted model's assumed data-generating process, by plugging in the maximum likelihood estimates of the parameters (based on the observed data).

```
mc_sim <- poLCA::poLCA.simdata(N=n,probs=mixOut$probs,P=mixOut$P)
mc_dat <- mc_sim$dat
colnames(mc_dat) <- colnames(mixOut$y)
```

c. Fit the latent class model to the simulated manifest variables, and calculate the maximum likelihood estimates (now based on the simulated data).

```
mc_fit <- poLCA(cbind(X1, X2, X3)~1, data=mc_dat, nclass=2)
```

d. The perturbed class membership probabilities can be readily obtained as the individual ("posterior") probabilities following Bayes' formula, using the parameter estimates from the simulated data, and the observed values of the manifest variables.

```
mc_dens <- poLCA::poLCA.posterior(lc=mc_fit,y=mixOut$y)
```

A.2 Model-based clustering

When the latent subgroups are measured by multiple continuous variables, a (model-based) clustering approach, such as a finite (multivariate) Gaussian mixture model [Fraley and Raftery, 2002] can be employed to characterize partitions with similar distributions of the manifest variables. The latent class model is parameterized by the components of a mixture distribution, such as the class-specific mean and covariance. We propose sampling the parameters using a similar parametric bootstrap approach to obtain the perturbed class membership probabilities. We describe the steps using the mclust package [Scrucca et al., 2016] for model-based clustering with Gaussian finite mixture models.

a. Fit the mixture model to the observed data.

```
# Xnames: names of manifest variables, e.g., c("X1", "X2", "X3")
# obs_data: observed dataset
mixOut <- densityMclust(data=obs_data[,Xnames],G=2)
```

b. Simulate a dataset under the fitted model's assumed data-generating process, by plugging in the maximum likelihood estimates of the parameters (based on the observed data).

```
mc_sim <- mclust::sim(modelName=mixOut$modelName,parameters=mixOut$
    parameters,n=n)
mc_dat <- data.frame(mc_sim[,-1]) # drop true classes in column 1
colnames(mc_dat) <- colnames(mixOut$data)
```

c. Fit the mixture model to the simulated manifest variables, and calculate the maximum likelihood estimates (now based on the simulated data).

```
mc_fit <- densityMclust(data=mc_dat,G=2)
```

d. The perturbed class membership probabilities can be readily obtained as the individual conditional probabilities of belonging to each mixture component, using the parameter estimates from the simulated data, and the observed values of the manifest variables.

```
mc_dens <- predict.densityMclust(object=mc_fit,newdata=mixOut$data,
   what="z")
```

B Monte Carlo Simulation Studies

We conducted Monte Carlo simulation studies to evaluate the operating characteristics of the proposed method. First, we assessed the ability of a constructed trajectory of the subgroup-specific effect estimates to recover the true subgroup-specific effect. Second, we compared the empirical coverages of the confidence intervals merely holding the estimated subgroup memberships fixed with the perturbed intervals that combined the CIs from the parametric bootstrap.

Furthermore, we evaluated the ability of an outcome model-based estimator to correct misclassification biases [Gardner, 2020]. Following Proposition 1 of Gardner [2020], the subgroup-specific average potential outcomes under the true memberships can be recovered from the average observed outcomes under the given subgroup memberships as follows:

$$\mathrm{E}\{Y(z)|\boldsymbol{x}, C^*\} = P_{z,\boldsymbol{x}}^{-1}\,\mathrm{E}(Y|z,\boldsymbol{x},C), \tag{1}$$

where $\mathrm{E}\{Y(z)|\boldsymbol{x}, C^*\}$, and $\mathrm{E}(Y|z,\boldsymbol{x},C)$, are both vectors of length $|\mathcal{C}|$ with the c-th elements $\mathrm{E}\{Y(z)|\boldsymbol{x}, C^* = c\}$, and $\mathrm{E}(Y|Z = z, \boldsymbol{X} = \boldsymbol{x}, \boldsymbol{C} = c)$, respectively. Let $P_{z,\boldsymbol{x}}$ denote the $|\mathcal{C}| \times |\mathcal{C}|$ matrix of classification probabilities, conditional on treatment $Z = z$ and covariates $\boldsymbol{X} = \boldsymbol{x}$, with the (j,k)-th entry being:

$$\Pr(C^* = k|C = j, z, \boldsymbol{x}) = \mathrm{E}\left\{ \frac{\lambda_k \mathrm{I}(C = j)}{\Pr(C = j|z,\boldsymbol{x})} \middle| Z = z, \boldsymbol{X} = \boldsymbol{x} \right\}, \tag{2}$$

where λ_k is the (predictive or posterior) probability of belonging to subgroup $k \in \mathcal{C}$. Let $\mathrm{E}\{Y(z)|C^* = c\} = \sum_{\boldsymbol{x}} \mathrm{E}\{Y(z)|\boldsymbol{x}, C^* = c\} \Pr(\boldsymbol{X} = \boldsymbol{x}|C^* = c)$ denote the average potential outcome marginalized over the distribution of the covariates $\boldsymbol{X}$ within the (true) subgroup

c, where

$$\Pr(\boldsymbol{X} = \boldsymbol{x}|C^* = c) = \frac{\Pr(\boldsymbol{X} = \boldsymbol{x}, C^* = c)}{\Pr(C^* = c)} = \frac{\mathrm{E}(\lambda_c|\boldsymbol{x})\Pr(\boldsymbol{X} = \boldsymbol{x})}{\mathrm{E}(\lambda_c)}.$$

The c-th subgroup-specific effect is then $\tau_c = \mathrm{E}\{Y(1)|C^* = c\} - \mathrm{E}\{Y(0)|C^* = c\}$. An estimator is obtained by plugging in an estimated outcome model $\hat{m}(z, \boldsymbol{x}, C = c)$ for $\mathrm{E}(Y|z, \boldsymbol{x}, C = c)$ given the imputed subgroups.

B.1 Study 1: Latent class analysis

We generated a single population of size $N = 10^5$ with two latent classes. Exactly $\Lambda \times N$ individuals were assigned to class 1 ($C_i^* = 1$), while the remaining $(1 - \Lambda) \times N$ individuals were assigned to class 2 ($C_i^* = 2$). The population proportions of individuals in class 1 and 2 were thus Λ and $1 - \Lambda$, respectively; we set $\Lambda = 0.4$. The covariates and potential outcomes for each individual in each class $c = 1, 2$ were then randomly generated as:

$$X_{di}|(C_i^* = c) \sim \text{Bernoulli}(\pi_c), d = 1, \ldots, 6;$$
$$Y_i(z)|(\boldsymbol{X}_i, C_i^* = c) = \text{Bernoulli}\left\{\text{expit}\left(\beta_{1c} z + \sum_{d=1}^{6} \beta_{d+1,c} X_{di}\right)\right\}, z = 0, 1.$$

The actual class-specific average treatment effects could then be calculated simply as the average difference in potential outcomes among individuals within each class; i.e.,

$$\left\{\sum_{i=1}^{N} \mathrm{I}(C_i^* = c)\right\}^{-1} \sum_{i=1}^{N} \mathrm{I}(C_i^* = c)\{Y_i(1) - Y_i(0)\}.$$

The dichotomous indicator variables, $X_{di}, d = 1, \ldots, 6$, were used to measure the latent class membership, with π_c being the probability of observing $X_{di} = 1$ among members of class c (whose $C_i^* = c$). We set $\pi_1 = 0.75$ and $\pi_2 = 0.25$ so that the response patterns between the two classes were sufficiently different. We set the class-specific treatment coefficients $\beta_{11} = -2.4$ and $\beta_{12} = 1.8$ so that they had opposite signs with a larger magnitude for class 1. The true class-specific population average treatment effects are displayed in Table 1. Ignoring

the possibility of heterogeneous treatment effects would lead to the erroneous conclusion that the treatment only slightly benefitted the population on average when there was a harmful average effect for a subpopulation. Each observed dataset of sample size $n = 1000$ was then generated as follows. First, exactly $\Lambda \times n$ individuals were randomly sampled (without replacement) from the subpopulation in class 1 ($C_i^* = 1$) of size $\Lambda \times N$, while the remaining $(1 - \Lambda) \times n$ individuals were randomly sampled (without replacement) from the subpopulation in class 2 ($C_i^* = 2$) of size $(1 - \Lambda) \times N$. Next, the observed treatments for the sampled individuals in each class $c = 1, 2$ were randomly generated as:

$$Z_i | (\boldsymbol{X}_i, C_i^* = c) \sim \text{Bernoulli} \left\{ \text{expit} \left(\sum_{d=1}^{6} \alpha_{dc} X_{di} \right) \right\}.$$

The observed outcomes were then revealed as $Y_i = Y_i(Z_i)$, so that the observed data for each individual were $(\boldsymbol{X}_i, Z_i, Y_i)$.

We considered two different settings for the roles of $\boldsymbol{X}_i$. First, we restricted $\boldsymbol{X}_i$ to only be indicators of the latent classes, by setting $\beta_{d+1,c} = \alpha_{dc} = 0$ for $d = 1, \ldots, 6; c = 1, 2$. Hence treatment was effectively randomized, and only the potential outcomes under treatment $Y(1)$ may have differed between the two classes. This setting was used to demonstrate that the outcome-based estimator of Gardner [2020] corrected for misclassification biases. Next, the indicators $\boldsymbol{X}_i$ were simultaneously common causes of treatment and outcome. For simplicity, we set the class-specific parameters in the outcome models as: $\beta_{21} = \beta_{31} = \beta_{41} = 0.7, \beta_{51} = \beta_{61} = \beta_{71} = 0$, and $\beta_{d+1,2} = 1 - \beta_{d+1,1}, d = 1, \ldots, 6$. Similarly, we set the class-specific parameters in the propensity score models as: $\alpha_{11} = \alpha_{21} = \alpha_{31} = 0.7, \alpha_{41} = \alpha_{51} = \alpha_{61} = 0$, and $\alpha_{d2} = 1 - \alpha_{d1}, d = 1, \ldots, 6$. Therefore, the confounders were non-overlapping covariate subsets between the two classes so that individuals who were misclassified could potentially be subject to inadequate control for confounding.

The individual class memberships were estimated by fitting a latent class model with two classes to the indicators $X_{di}, d = 1, \ldots, 6$, using the poLCA package in R. We calculated the

class-specific effect estimates and the 95% CIs either under the true (unknown) individual class memberships or holding the estimated individual class memberships fixed. To avoid conflating the biases due to misclassification with biases due to label switching, we simply compared the estimated and true class memberships in each sample, then relabelled the classes to maximize the similarity between the estimated and true class memberships, and subsequently, the number of individuals who would be correctly classified. For each latent class $c \in \mathcal{C}$ in turn, we constructed a trajectory of the subgroup-specific effect estimates based on the estimated class membership probabilities following the procedure described in the main text. To account for the uncertainty in the estimated class membership probabilities, we then calculated the class-specific effect estimates and the 95% CIs using 100 perturbed probabilities following the procedure described in the main text. We combined the separate CIs to construct the perturbed CI across all perturbations.

B.2 Study 2: Gaussian mixture distribution

In this study, the latent subgroups were determined by the components of a multivariate Gaussian mixture distribution for the covariates. The data-generating process in this study differed from that of the previous study only in that the covariates (and possibly manifest indicators of the latent subgroups) $\boldsymbol{X}_i$ were randomly drawn from a multivariate normal distribution:

$$\begin{aligned}
\boldsymbol{X}_i|(C_i^* = c) &\sim \mathcal{N}_6(\boldsymbol{\mu}_c, \Sigma_c), \\
\boldsymbol{\mu}_1 &= (0.75, 0.75, 0.75, 0.25, 0.25, 0.25)^T, \\
\boldsymbol{\mu}_2 &= -\boldsymbol{\mu}_1, \\
\Sigma_c &= \boldsymbol{I}_6, c = 1, 2.
\end{aligned}$$

The (subgroup-specific) mean and covariance are denoted by $\boldsymbol{\mu}_c$ and Σ_c, respectively. For simplicity, we used the same values as study 1 for all parameters in the treatment and

outcome data-generating models. The observed datasets were generated using the same procedure. In each observed sample, the individual subgroup memberships were estimated by fitting a multivariate Gaussian finite mixture model with two subgroups (components) to the covariates $\boldsymbol{X}_i$, using the mclust package Scrucca et al. [2016] in R. All other aspects of the simulation study were maintained.

B.3 Results

The results for 1000 replications under each setting are presented below in Tables 1 and 2. First, the constructed trajectories of point estimates recovered the true class-specific effects between 50% to 99% of the time empirically. The empirical probabilities were lowest (50-60%) for one of the classes when the latent class indicators were simultaneously confounders of treatment and outcome. For the other class, the constructed trajectories intersected the true class-specific effect in more than 90% of the simulated datasets across all settings.

Second, the estimators were unbiased under the true class memberships (which are unknown in practice), and the 95% CIs achieved the nominal coverage level empirically across all settings. Under the estimated class memberships, both estimators that either held the class memberships fixed or used perturbed membership probabilities suffered from misclassification biases across all settings as expected. However, the 95% CIs holding the estimated class memberships fixed had empirical coverages (far) below their nominal level. The perturbed CIs improved the empirical coverages but fell short of the nominal level. The bias-corrected estimators (partially) removed biases due to misclassification only when the indicators were not simultaneously confounders of treatment and outcome. Otherwise, the biases could be greater (in magnitude) than those based simply on the estimated class memberships.

Table 1: Summaries of the constructed trajectories of point estimates for the class-specific effects in the simulation studies. The proportion of individuals in class 1 was Λ. The latent class indicators were either not confounders ("No") or simultaneously confounders ("Yes") of treatment and outcome. The trajectories were constructed using estimated class membership probabilities from either a latent class analysis ("LCA"; study 1) or Gaussian mixture model ("GMM"; study 2).

Classes	Λ	Confounders	Recovery		Coverage		Point estimates				True values	
		Class	1	2	1	2	1 (min.)	1 (max.)	2 (min.)	2 (max.)	1	2
LCA	0.40	No	0.91	0.94	1.00	1.00	-0.61	0.06	0.00	0.57	-0.42	0.36
LCA	0.40	Yes	0.49	0.92	0.99	0.99	-0.21	0.03	-0.06	0.17	-0.21	0.06
GMM	0.40	No	0.92	0.98	1.00	1.00	-0.61	0.09	-0.06	0.58	-0.41	0.35
GMM	0.40	Yes	0.59	0.99	1.00	1.00	-0.45	0.25	-0.06	0.66	-0.43	0.33

Note. The average proportions of simulated datasets where the constructed trajectory recovered the true class-specific effect in each class are stated ('Recovery'). The average proportions of simulated datasets where the pointwise 95% confidence band of the constructed trajectory captured the true class-specific effect in each class are stated ('Coverage'). The average minimum ('min.') and maximum ('max.') point estimates in the constructed trajectory for each class across simulated datasets are presented. All results were rounded to two decimal places.

Table 2: Summaries of the point estimates, and coverage of the 95% CIs, for the class-specific effects in the simulation studies. The proportion of individuals in class 1 was Λ. The latent class indicators were either not confounders ("No") or simultaneously confounders ("Yes") of treatment and outcome. The individual class memberships used to separate the individuals were either the true unknown values, or estimated using either a latent class analysis ("LCA"; study 1) or Gaussian mixture model ("GMM"; study 2) then held fixed, or based on perturbed probabilities that accounted for the uncertainty in the estimates. Either an augmented inverse propensity score weighted (AIPW) estimator or the outcome model-based bias-corrected estimator of Gardner [2020] was utilized within each subgroup.

Classes	Λ	Confounders	CI type	Estimator	Bias		Coverage		ESE		RMSE		Prop. Correct	
				Class	1	2	1	2	1	2	1	2	1	2
LCA	0.40	No	True	AIPW	0.07	0.21	0.94	0.95	4.29	3.54	4.29	3.55	1.00	1.00
LCA	0.40	No	Fixed	AIPW	6.39	-7.56	0.68	0.44	5.13	3.74	8.20	8.44	0.74	0.83
LCA	0.40	No	Perturbed	AIPW	6.39	-7.56	0.81	0.71	5.13	3.74	8.20	8.44	0.74	0.84
LCA	0.40	No	–	Bias corrected	-1.65	0.03	–	–	6.03	4.38	6.25	4.38	–	–
LCA	0.40	Yes	True	AIPW	0.46	-1.58	0.84	0.80	6.50	3.33	6.52	3.69	1.00	1.00
LCA	0.40	Yes	Fixed	AIPW	4.25	-2.42	0.86	0.77	7.12	4.46	8.29	5.08	0.74	0.83
LCA	0.40	Yes	Perturbed	AIPW	4.25	-2.42	0.94	0.87	7.12	4.46	8.29	5.08	0.74	0.84
LCA	0.40	Yes	–	Bias corrected	-4.47	7.01	–	–	12.04	9.19	12.85	11.56	–	–
GMM	0.40	No	True	AIPW	0.57	-0.36	0.92	0.95	4.30	3.47	4.33	3.49	1.00	1.00
GMM	0.40	No	Fixed	AIPW	9.13	-6.26	0.44	0.60	5.79	4.19	10.81	7.53	0.75	0.85
GMM	0.40	No	Perturbed	AIPW	9.13	-6.26	0.82	0.88	5.79	4.19	10.81	7.53	0.75	0.85
GMM	0.40	No	–	Bias corrected	-0.84	1.72	–	–	7.54	4.60	7.59	4.91	–	–
GMM	0.40	Yes	True	AIPW	0.21	-0.20	0.92	0.92	5.00	4.54	5.01	4.54	1.00	1.00
GMM	0.40	Yes	Fixed	AIPW	12.33	-2.79	0.37	0.81	7.74	5.08	14.56	5.80	0.75	0.85
GMM	0.40	Yes	Perturbed	AIPW	12.33	-2.79	0.81	0.96	7.74	5.08	14.56	5.80	0.75	0.85
GMM	0.40	Yes	–	Bias corrected	13.74	1.71	–	–	7.22	5.18	15.52	5.45	–	–

Note. All effect estimates were multiplied by 100 to improve readability. The empirical SE ("ESE") of the effect estimates (i.e., standard deviation around the average over all simulated datasets), and root mean squared error ("RMSE") of the effect estimates (calculated as the square root of the mean of squares of the estimates centered at the true value), are presented. The average proportions of correctly classified individuals across all simulated datasets are stated. A dashed line ("–") indicates a non-applicable setting. The true population class-specific effects are displayed in Table 1. All results were rounded to two decimal places.

C Perturbed pointwise confidence bands for the trajectory

Pointwise confidence bands for the trajectory of the subgroup-specific effect in a certain subgroup $c \in \mathcal{C}$ can be readily constructed using the perturbed probabilities as follows. Given a set of perturbed subgroup membership probabilities $(\tilde{\lambda}_{ic}, i = 1, \ldots, n)$, construct the trajectory as described in the main text using $\tilde{\lambda}_{ic}$ in place of $\widehat{\lambda}_{ic}$. Denote the resulting sequence of (nested) partitions of individuals by $\{\tilde{\mathcal{S}}_c(j) : j = \tilde{m}_c^*, \ldots, \tilde{m}_c^{**}\}$, where $\tilde{m}_c^* = \sum_{i=1}^n \mathrm{I}(\tilde{\lambda}_{ic} \geq 0.99)$ and $\tilde{m}_c^{**} = \sum_{i=1}^n \mathrm{I}(\tilde{\lambda}_{ic} \geq 0.01)$ are determined by the user-specified thresholds. For each (nested) partition of individuals $\{\tilde{\mathcal{S}}_c(j) : j = \tilde{m}_c^*, \ldots, \tilde{m}_c^{**}\}$, calculate the 95% CI for the subgroup-specific effect. Therefore, the resulting sequence of subgroup-specific effect estimates is based on an increasing number of individuals (ordered by their perturbed membership probabilities and added one at a time) belonging to that subgroup c. Repeating these steps for repeated perturbations yields a collection of trajectories for the CI. The pointwise confidence band can then be determined using the union method across all perturbations.

D Average indicator values by latent class in the applied examples

We report the average value of each of the manifest indicators for the RHC data used in the applied examples. These values indicated the probability (if the indicator was binary) or quintile (if the indicator was discretized) that a patient representative of that class would exhibit for that characteristic.

Table 3: **Average value for each manifest indicator used in the measurement model by latent class for the RHC data. The p-value from a Chi-squared test of the frequency table of class membership versus values of each indicator is displayed in the rightmost column. The estimated proportion in each class is stated in the last row. All results were rounded to three decimal places.**

Indicator	Class 1	Class 2	Class 3	Class 4	p-value
Primary disease category: COPD (cat1_copd)	0.018	0.003	0.225	0.007	0.000
Primary disease: MOSF with Sepsis (cat1_mosfsep)	0.145	0.000	0.049	0.427	0.000
Primary disease category: MOSF with Malignancy (cat1_mosfmal)	0.342	0.000	0.001	0.000	0.000
Primary disease: CHF (cat1_chf)	0.008	0.920	0.005	0.008	0.000
Primary disease category: Coma (cat1_coma)	0.033	0.000	0.175	0.032	0.000
Primary disease category: Cirrhosis (cat1_cirr)	0.016	0.002	0.000	0.091	0.000
Primary disease category: Lung Cancer (cat1_lung)	0.031	0.002	0.001	0.000	0.000

Continued on next page

Table 3 Continued from previous page

Indicator	Class 1	Class 2	Class 3	Class 4	p-value
Primary disease category: Colon Cancer (cat1_colon)	0.004	0.004	0.000	0.000	0.000
Secondary disease category: MOSF with Sepsis (cat2_mosfsep)	0.232	0.012	0.062	0.193	0.000
Secondary disease category: Coma (cat2_coma)	0.004	0.000	0.030	0.013	0.000
Secondary disease category: MOSF with Malignancy (cat2_mosfmal)	0.195	0.000	0.002	0.000	0.000
Secondary disease category: Lung Cancer (cat2_lung)	0.013	0.000	0.000	0.000	0.000
Secondary disease category: Cirrhosis (cat2_cirr)	0.003	0.000	0.000	0.015	0.000
Secondary disease: Colon Cancer (cat2_colon)	0.002	0.000	0.000	0.000	0.049
Respiratory Diagnosis (resp)	0.404	0.088	0.520	0.283	0.000
Cardiovascular Diagnosis (card)	0.220	0.927	0.305	0.303	0.000
Neurological Diagnosis (neuro)	0.070	0.007	0.275	0.044	0.000
Gastrointestinal Diagnosis (gastr)	0.183	0.012	0.035	0.291	0.000
Renal Diagnosis (renal)	0.077	0.013	0.002	0.086	0.000
Metabolic Diagnosis (meta)	0.043	0.022	0.027	0.068	0.000
Hematologic Diagnosis (hema)	0.212	0.011	0.007	0.040	0.000
Sepsis Diagnosis (seps)	0.259	0.000	0.034	0.294	0.000
Trauma Diagnosis (trauma)	0.000	0.000	0.013	0.012	0.000
Orthopedic Diagnosis (ortho)	0.001	0.002	0.001	0.001	0.910
Duke Activity Status Index (das2d3pc)	1.809	1.447	2.188	1.998	0.000
Do Not Resuscitate (dnr1)	0.168	0.056	0.124	0.090	0.000
Cancer: Yes (ca_yes)	0.681	0.061	0.083	0.000	0.000

Continued on next page

Table 3 Continued from previous page

Indicator	Class 1	Class 2	Class 3	Class 4	p-value
Cancer: Metastatic (ca_meta)	0.314	0.016	0.007	0.000	0.000
Support model estimate of the prob. of surviving 2 months (surv2md1)	0.718	3.701	2.434	1.956	0.000
APACHE score (aps1)	2.525	0.737	1.051	2.655	0.000
Glasgow Coma Score (scoma1)	0.425	0.018	0.664	0.536	0.000
Weight in kilograms (wtkilo1)	1.969	2.298	1.812	2.100	0.000
Temperature (temp1)	2.033	0.842	2.039	2.002	0.000
Mean blood pressure (meanbp1)	1.609	2.287	2.558	1.627	0.000
Respiratory Rate (resp1)	2.205	1.615	1.850	1.931	0.000
Heart rate (hrt1)	2.375	1.199	1.736	2.104	0.000
PaO2/FIO2 ratio (pafi1)	1.804	2.773	1.920	1.833	0.000
PaCo2 (paco21)	1.710	1.908	2.211	1.508	0.000
Arterial PH (ph1)	1.790	2.322	2.046	1.843	0.000
White Blood Cell Count (wblc1)	1.670	1.116	2.012	2.316	0.000
Hematocrit (hema1)	1.385	3.052	2.742	1.468	0.000
Sodium (sod1)	1.764	1.478	2.015	1.839	0.000
Potassium (pot1)	1.949	2.163	1.813	1.990	0.000
Creatinine (crea1)	2.021	2.037	1.206	2.496	0.000
Bilirubin (bili1)	1.371	1.035	0.862	1.605	0.000
Albumin (alb1)	1.243	2.128	1.894	1.121	0.000
Urine output (urin1)	3.624	4.038	3.709	3.367	0.000

Continued on next page

Table 3 Continued from previous page

Indicator	Class 1	Class 2	Class 3	Class 4	p-value
Acute MI, Peripheral Vascular Disease, Severe Cardiovascular Symptoms with NYHA-Class III, Very Severe Cardiovascular Symptoms with NYHA-Class IV (cardiohx)	0.076	0.745	0.130	0.151	0.000
Congestive Heart Failure (chfhx)	0.070	0.849	0.138	0.131	0.000
Dementia, Stroke or Cerebral Infact, Parkinson's Disease (dementhx)	0.054	0.069	0.128	0.103	0.000
Psychiatric History, Active Psychosis or Severe Depression (psychhx)	0.041	0.037	0.093	0.065	0.000
Chronic Pulmonary Disease, Severe Pulmonary Disease, Very Severe Pulmonary Disease (chrpulhx)	0.134	0.165	0.333	0.106	0.000
Chronic Renal Disease, Chronic Hemodialysis or Peritoneal Dialysis (renalhx)	0.023	0.030	0.007	0.089	0.000
Cirrhosis, Hepatic Failure (liverhx)	0.028	0.006	0.001	0.160	0.000
Upper GI Bleeding (gibledhx)	0.010	0.002	0.000	0.076	0.000
Solid Tumor, Metastatic Disease, Chronic Leukemia/Myeloma, Acute Leukemia, Lymphoma (malighx)	0.963	0.076	0.089	0.000	0.000
Immunosupperssion, Organ Transplant, HIV Positivity, Diabetes Mellitus Without End Organ Damage, Diabetes Mellitus With End Organ Damage, Connective Tissue Disease (immunhx)	0.244	0.329	0.197	0.329	0.000
Transfer from Another Hospital (transhx)	0.060	0.124	0.126	0.133	0.000

Continued on next page

Table 3 Continued from previous page

Indicator	Class 1	Class 2	Class 3	Class 4	p-value
Definite Myocardial Infarction (amihx)	0.029	0.042	0.044	0.029	0.008
Weight is zero (wt0)	0.103	0.021	0.136	0.059	0.000
Proportion in each class	0.20	0.32	0.08	0.39	NA

End of table

References

J. G. Dias and J. K. Vermunt. Bootstrap methods for measuring classification uncertainty in latent class analysis. In A. Rizzi and M. Vichi, editors, *Compstat 2006 - Proceedings in Computational Statistics*, pages 31–41, Heidelberg, 2006. Physica-Verlag HD. ISBN 978-3-7908-1709-6. doi: 10.1007/978-3-7908-1709-6_3.

C. Fraley and A. E. Raftery. Model-based clustering, discriminant analysis, and density estimation. *Journal of the American Statistical Association*, 97(458):611–631, 2002. doi: 10.1198/016214502760047131.

J. Gardner. Identification and estimation of average causal effects when treatment status is ignorable within unobserved strata. *Econometric Reviews*, 39(10):1014–1041, 2020. doi: 10.1080/07474938.2020.1735748. URL `https://doi.org/10.1080/07474938.2020.1735748`.

D. A. Linzer, J. B. Lewis, et al. poLCA: An R package for polytomous variable latent class analysis. *Journal of statistical software*, 42(10):1–29, 2011. doi: 10.18637/jss.v042.i10.

L. Scrucca, M. Fop, T. B. Murphy, and A. E. Raftery. mclust 5: clustering, classification and density estimation using Gaussian finite mixture models. *The R journal*, 8(1):289, 2016. doi: 10.32614/RJ-2016-021.
